# USP28 Promotes Osimertinib Resistance in H1975 NSCLC Cells by Deubiquitinating and Stabilizing SIRT1

**DOI:** 10.1002/kjm2.70095

**Published:** 2025-08-26

**Authors:** Hu‐Sen Fan, Xiu‐Mei Li, Jia‐Qi Gu, Hai‐Feng Wang, Zhen‐Jiang Zhang

**Affiliations:** ^1^ Department of Thoracic Surgery Weifang People's Hospital Weifang China; ^2^ Thoracic Surgery Medical Center Weifang People's Hospital Weifang China; ^3^ Shanghai Renxin Medical Laboratory Co., Ltd Shanghai China

**Keywords:** H1975/OSI cells, NSCLC, osimertinib resistance, USP28 deubiquitination

## Abstract

Osimertinib (OSI) resistance in non‐small cell lung cancer (NSCLC) remains a significant challenge. This report explored the precise role of USP28 on OSI resistance in NSCLC and identified a functional downstream effector. OSI‐resistant H1975 cells (H1975/OSI) were established by long‐term OSI exposure. USP28 and SIRT1 expression levels were analyzed by quantitative PCR, immunoblotting, and immunohistochemistry. Functional assays included cell viability, colony formation, EdU incorporation, apoptosis analysis, and glycolysis assays. The interaction between USP28 and SIRT1 was confirmed by co‐immunoprecipitation (Co‐IP) assay and SIRT1 protein stability analysis. In vivo validation was performed using H1975/OSI xenograft models. USP28 and SIRT1 were upregulated in H1975/OSI cells and OSI‐resistant NSCLC tissues. USP28 overexpression enhanced cell proliferation and glycolysis, suppressed apoptosis, and conferred OSI resistance in H1975 cells, while its depletion exerted opposite effects in H1975/OSI cells. Mechanistically, USP28 stabilized SIRT1 by deubiquitination. SIRT1 knockdown attenuated the effects of USP28 overexpression, while SIRT1 restoration reversed the phenotype alterations upon USP28 depletion. In vivo, USP28 depletion sensitized H1975/OSI xenografts to OSI treatment. Our study indicates that USP28 promotes OSI resistance in NSCLC by deubiquitinating SIRT1. Targeting the USP28/SIRT1 axis may represent a novel therapeutic approach to overcome OSI resistance in EGFR‐mutant NSCLC.

## 1 Introduction

Non‐small cell lung cancer (NSCLC) is composed of a heterogeneous group of diseases [1], and as the most common pathologic type of lung cancer, it is also the leading cause of cancer‐related deaths worldwide. Characterized by molecular heterogeneity and aggressive progression, NSCLC poses significant therapeutic challenges, particularly in advanced stages [2, 3]. The advent of precision medicine has revolutionized NSCLC management, with targeted therapies emerging as a cornerstone for patients harboring actionable genetic alterations [4]. The development of third‐generation EGFR tyrosine kinase inhibitors (EGFR‐TKIs), such as osimertinib (OSI) [5], marked a paradigm shift in treating EGFR mutation‐positive NSCLC. OSI has demonstrated superior efficacy over earlier‐generation TKIs and has become the standard first‐line option [6, 7, 8]. Despite these advances, acquired resistance to OSI remains inevitable, observed in nearly all patients within 12–24 months, underscoring a critical barrier to long‐term disease control [9]. Therefore, the complexity of OSI resistance necessitates further exploration of molecular drivers and adaptive signaling pathways to develop tailored therapeutic approaches.

Ubiquitination, a crucial post‐translational modification [10], regulates protein stability, degradation, and function, with deubiquitinases (DUBs) counteracting this process by removing ubiquitin chains [11]. Among DUBs, ubiquitin‐specific protease 28 (USP28) has emerged as a critical regulator of oncogenic pathways [12]. USP28 stabilizes key oncoproteins, such as c‐MYC, c‐JUN, and TCF7L2, by preventing their proteasomal degradation, thereby promoting tumorigenesis in various cancers, including breast cancer and hepatocellular carcinomas [13, 14]. Notably, USP28 exhibits context‐dependent roles, acting as both an oncogene and tumor suppressor across cancer types, which underscores its complex regulatory mechanisms in cellular proliferation, DNA repair, and metastasis [15]. Furthermore, a substantial body of research highlights that USP28 contributes to drug resistance in cancer [16, 17]. In NSCLC, USP28 has been identified as a potential driver of oncogenesis, contributing to tumor growth and progression through the stabilization of specific substrates involved in cell proliferation and survival pathways [18, 19]. However, no reports explored the involvement of USP28 in OSI resistance in NSCLC.

Sirtuin 1 (SIRT1), an NAD^+^‐dependent deacetylase, is a critical regulator of cellular homeostasis, metabolism, and stress response, thereby participating in carcinogenesis [20]. In NSCLC, SIRT1 exhibits dual roles, acting as both an oncogenic driver and anti‐tumor factor depending on the context. For instance, silencing of SIRT1 impedes NSCLC development through the suppression of cell growth and metastasis [21]. Conversely, SIRT1 can exert a tumor suppressive role in K‐RAS‐driven NSCLC by modulating cell apoptosis and extracellular matrix organization [22]. Despite these advances, the full spectrum of SIRT's functions in NSCLC, particularly in the context of OSI resistance, remains to be fully elucidated.

This study investigated the precise action of USP28 on OSI resistance in NSCLC, finding that USP28 can confer OSI resistance in H1975 NSCLC cells. Further, our study identified a functional downstream effector of USP28 in the context. These findings advance our understanding of the molecular underpinnings of OSI resistance in EGFR‐mutant NSCLC and highlight USP28 as a potential therapeutic node for resistance mitigation.

## 2 Materials and Methods

### 2.1 Bioinformatics Analysis

In order to search for the factors associated with OSI resistance in NSCLC, this study performed comparative transcriptome analysis between OSI‐resistant H1975 cells (H1975/OSI) and their sensitive parental counterparts H1975 using the high‐throughput sequencing data from the GSE236654 dataset (available at NCBI GEO: https://www.ncbi.nlm.nih.gov/geo/query/acc.cgi?acc=GSE236654). Differential gene expression analysis was conducted with stringent screening criteria (|Fold‐Change| ≥ 1.5 and adjusted *P*‐value < 0.05) to identify significantly dysregulated genes associated with acquired resistance. The online comprehensive Ubibrowser2.0 database was utilized to predict the potential substrates of USP28 at http://ubibrowser.bio‐it.cn/ubibrowser_v3/home/index. The UALCAN algorithm (https://ualcan.path.uab.edu/cgi‐bin/CPTAC‐Result.pl?genenam=SIRT1&ctype=LUA) was applied to analyze the expression pattern of SIRT1 in NSCLC tumors compared with normal counterparts.

### 2.2 Cell Culture and Establishment of H1975/OSI Cell Line

H1975 NSCLC cells (Cat No. IM‐H119) were obtained from Immocell (Xiamen, China), which had been confirmed by STR authentication. Cell culture was performed at 37°C in single‐use culture flasks (Corning, Shanghai, China) using the complete growth medium consisting of DMEM (Procell, Wuhan, China), 1% penicillin/streptomycin (Beyotime, Shanghai, China), and 10% FBS (Life Technologies, Abingdon, UK). The 293 T cell line (Cat No. IM‐H222) was obtained from Immocell and the cells were cultivated using Immocell‐developed protocols. The H1975/OSI cell line was generated through intermittent induction by culturing H1975 cells in low concentrations (0.03–1.5 μM) of OSI (Selleck, Shanghai, China) as described previously [23]. In brief, NCI‐H1975 cells were initially exposed to 0.03 μM OSI for 72 h, followed by recovery in drug‐free medium until surviving cells resumed normal proliferation. Through iterative cycles of stepwise concentration escalation (0.03–1.5 μM) over about 6 months, stable OSI‐resistant populations were established. For the maintenance of resistance phenotypes, H1975/OSI cells were grown in media containing 0.2 μM of OSI.

### 2.3 Plasmids, shRNAs, and Lentivirus Constructs

Plasmids, including pLV2‐CMV‐USP28(human)‐Puro (oeUSP28), pCMV‐SIRT1(human)‐Neo (oeSIRT1), and corresponding plasmid control (oeNC), were obtained from Miaoling Biology (Wuhan, China). The 1:1:1 ratio mixture of pLV3‐U6‐USP28(human)‐shRNA1‐EF1a‐Puro (Miaoling Biology), pLV3‐U6‐USP28(human)‐shRNA2‐EF1a‐Puro (Miaoling Biology), and pLV3‐U6‐USP28(human)‐shRNA3‐EF1a‐Puro (Miaoling Biology) was used as the shRNA targeting USP28 (shUSP28). The 1:1:1 ratio mixture of pLV3‐U6‐SIRT1(human)‐shRNA1‐EGFP‐Puro, pLV3‐U6‐SIRT1(human)‐shRNA2‐EGFP‐Puro, and pLV3‐U6‐SIRT1(human)‐shRNA3‐EGFP‐Puro, all of which were procured from Miaoling Biology, was defined as the SIRT1‐shRNA (shSIRT1). The nontarget shRNA control (shNC) served as the negative counterpart. Based on the shUSP28 or shNC construct, lentivirus particles expressing shUSP28 or shNC were generated by Genomeditech (Shanghai, China).

### 2.4 Transfection and Transduction

As described by the supplier (Life Technologies), transient transfection of oeNC, oeUSP28, or oeUSP28 + shSIRT1 was conducted into H1975 cells and shNC, shUSP28, or shUSP28 + oeSIRT1 into H1975/OSI cells using Lipofectamine 3000. Briefly, cells were incubated with transfection complexes for 6 h at 37°C, refreshed with the complete medium, and maintained in culture for 24–72 h post‐transfection. For the generation of a stable USP28 silencing cell line, H1975/OSI cells were transduced with MOI‐optimized shUSP28 lentiviral particles and selected with puromycin (1 μg/mL) for at least 2 weeks as previously described [24]. Parallel cultures transduced with sh‐NC lentivirus served as negative controls.

### 2.5 Cell Cytotoxicity Assay

The in vitro cytotoxicity of OSI was assessed using a CCK‐8 assay (MedChemExpress, Shanghai, China) according to standardized protocols. Briefly, both untransfected and transfected cells were seeded in 96 multi‐well culture plates and exposed to gradient concentrations of OSI for 24 h. Cellular viability was subsequently quantified through a 3‐h incubation with CCK‐8 reagent, with optical density measurements at 450 nm using a Viktor X3 reader (Perkin Elmer, Turku, Finland). Dose–response curves were generated by plotting cell viability percentages against corresponding OSI concentrations, from which the IC50 value was mathematically derived.

### 2.6 Human Specimens and Ethic Statement

This retrospective study utilized surgicopathologically confirmed NSCLC specimens obtained from Weifang People's Hospital. Cohort stratification included: (1) 35 recurrent NSCLC patients after treatment based on OSI‐based therapy (resistant group); (2) 22 treatment‐naive NSCLC patients who maintained disease control with OSI‐based treatment (sensitive group). OSI‐based therapy consisted of OSI (80 mg once daily) plus bevacizumab (7.5–15 mg/kg administered every 3 weeks). These patients received OSI‐based therapy for a mean duration of 27.6 months, and NSCLC recurrence was observed between 20 and 36 months (mean 25.8 ± 4.8 months) following treatment. All participants provided informed consent prior to specimen acquisition. All samples were subjected to RNA extraction for USP28 mRNA expression analysis. For protein expression analysis, five OSI‐resistant samples and five sensitive samples were selected with USP28 mRNA expression levels closest to their respective group means. Experimental protocols strictly adhered to Declaration of Helsinki guidelines, with ethical oversight maintained through Weifang People's Hospital institutional review board.

### 2.7 Analysis of USP28 and SIRT1 mRNA Expression

Total RNA was extracted from cultivated cells (5 × 10^6^) or clinical specimens (60 mg) using the BeyoMag RNA Kit (Beyotime) following the vendor's specifications. Synthesis of first‐strand cDNA was conducted with the QuantiTect RT Kit (Qiagen, Frankfurt, Germany) under standardized reverse transcription conditions. SYBR Green‐based quantitative PCR (Qiagen) was performed in triplicate on a Rotorgene 6000 machine (Qiagen) using gene‐specific primers (Table 1). Relative quantification was calculated using the 2^−ΔΔCt^ method with β‐actin as an endogenous control.

**TABLE 1:** Primers sequences used for qPCR.

Name		Primers for PCR (5′‐3′)
USP28	Forward	CACAGGCATTCAGGACCCTT
	Reverse	ACTTCCTTGTTGGCAGCACT
SIRT1	Forward	TGGGTACCGAGATAACCTTCT
	Reverse	TGCCAATCATAAGATGTTGCTG
β‐Actin	Forward	CTTCGCGGGCGACGAT
	Reverse	CCACATAGGAATCCTTCTGACC

### 2.8 Subcutaneous Xenograft Models and Immunohistochemical Assay

All procedures complied with ARRIVE guidelines and were conducted under Weifang People's Hospital Institutional Animal Care and Use Committee. Fifteen 6‐week‐old male BALB/c nude mice (Vital River Laboratory, Beijing, China) were randomized into three cohorts through stratified allocation: shNC+vehicle (*n* = 5), shNC+OSI (*n* = 5), and shUSP28 + OSI (*n* = 5). H1975/OSI cells (1 × 10^7^) cells in 200 μL Matrigel/PBS (3:1 v/v) pre‐transduced with either shUSP28 lentivirus or control particles were implanted via subcutaneous injection into the right dorsal flank. Digital caliper measurements obtained every 5 days determined tumor volume using the modified formula: (longest diameter × (shortest diameter)^2^/2). At tumor volume reaching ~100 mm^3^, administration of OSI (20 mg/kg/d) or vehicle began via gavage and was performed every 2 days. At experimental termination (day 20 post‐implantation), xenografts were excised under aseptic conditions for weight measurement and expression analysis. Immunohistochemical assay was conducted with paraformaldehyde‐fixed and paraffin‐embedded tissue sections. Serial 4‐μm sections were prepared, and immunostaining was performed using rabbit monoclonal to Ki‐67 (Cat No. A20018, ABclonal, Wuhan, China, 1 to 500) or rabbit polyclonal to cleaved‐caspase 3 (Cat No. 25128–1‐AP, Proteintech, Wuhan, China, 1 to 300). Signal visualization employed: HRP‐labeled IgG secondary antibody, DAB chromogen development, and hematoxylin counterstaining.

### 2.9 Immunoblotting

Using the Total Protein Extraction Kit (Abcam, Cambridge, UK) as per the accompanying guidelines, protein samples were prepared from cultivated cells (3 × 10^7^) or clinical specimens (200 mg). Protein samples (30 μg per lane) were resolved through SDS‐PAGE and electrophoretically transferred to nitrocellulose membranes (0.45 μm pore size; Beyotime). Membranes were incubated overnight at 4°C with the following antibodies: rabbit polyclonal to USP28 (Cat No. 17707–1‐AP, Proteintech, 1 to 12,000), rabbit polyclonal to GLUT1 (Cat No. 21829–1‐AP, Proteintech, 1 to 20,000), rabbit polyclonal to HK2 (Cat No. 22029–1‐AP, Proteintech, 1 to 20,000), rabbit monoclonal to SIRT1 (Cat No. ab189494, Abcam, 1 to 1000), rabbit monoclonal to ubiquitin (Ub, Cat No. ab134953, Abcam, 1 to 5000), or rabbit polyclonal to β‐actin (Cat No. 20536–1‐AP, Proteintech, 1 to 8000). After washing, the anti‐rabbit HRP‐conjugated secondary antibody was applied for 1 h at room temperature. Protein bands were detected using an EZ‐ECL Kit (Biological Industries, Beit‐Haemek, Israel) and imaged with an Amersham 600 imager (GE Healthcare Life Sciences, Little Chalfont, UK).

### 2.10 Colony Formation Assay

Following transfection, H1975 and H1975/OSI cells were plated in 6‐well Corning Costar plates, which were cultured in 10% FBS DMEM under controlled conditions. After 12–14 days of undisturbed proliferation with medium renewal every 72 h, colonies were stained with 0.5% crystal violet and quantified using ImageJ (NIH, Bethesda, MD, USA), applying rigorous thresholds (≥ 0.5 mm^2^ area).

### 2.11 EdU Incorporation Assay

A Click‐iT EdU‐594 Assay Kit (Servicebio, Wuhan, China) was applied for the assessment of cell proliferation. At 48 h post‐transfection, H1975 and H1975/OSI cells were incubated with 10 μM EdU working solution. Fluorescent detection was achieved using Click‐iT iF594 azide (showing a red fluorescence) for 30 min, followed by nucleus staining with 5 μg/mL DAPI (showing a blue fluorescence) for 10 min. Quantitative analysis of EdU^+^ cells was performed using a TCS SP5 confocal system equipped with LAS X Navigator (Leica, Wetzlar, Germany) across 5 randomized fields per sample.

### 2.12 Flow Cytometry

To assess apoptosis, H1975 and H1975/OSI cells were dual‐stained with FITC‐Annexin V and propidium iodide (PI) 72 h post‐transfection using a commercial Apoptosis Detection Kit as suggested by the supplier (BD Biosciences, Heidelberg, Germany). Cellular analysis was conducted with a CytoFLEX SRT flow cytometer (Beckman Coulter, Krefeld, Germany), and the proportion of late apoptotic cells (Annexin V^+^/PI^+^) was quantified.

### 2.13 Determination of Glucose Uptake and Lactate Production Levels

After the indicated transfection, H1975 and H1975/OSI cells were harvested for the evaluation of metabolic parameters. In accordance with the manufactory protocols, the glucose uptake levels were quantified using the Enhanced Glucose Assay Kit (Beyotime), and the lactate secretion was detected with the Lactate Assay Kit (Abcam). Data acquisition utilized the BioTek Synergy H1 Hybrid Reader (Bio‐Tek Instruments, Bad Friedrichshall, Germany).

### 2.14 Immunoprecipitation and Co‐IP Assays

For these assays, a commercially available IP Assay Kit from Beyotime was employed. Following the manufacturer guidelines, lysates of untransfected or transfected H1975 and H1975/OSI cells were prepared and added into Protein A magnetic beads previously pre‐incubated with rabbit polyclonal to USP28 (Cat No. 17707–1‐AP, Proteintech), rabbit monoclonal to SIRT1 (Cat No. ab189494, Abcam), or rabbit monoclonal to IgG (Cat No. ab172730, Abcam). After a 6‐h incubation at 4°C, immunoprecipitates were isolated followed by elution of bound proteins through 5‐min boiling in SDS loading buffer, enabling subsequent evaluation of the enrichment levels of USP28, SIRT1, or ubiquitinated SIRT1.

### 2.15 Analysis of SIRT1 Protein Stability

H1975 cells subjected to oeNC or oeUSP28 transfection and shNC‐ or shUSP28‐transfected H1975/OSI cells were exposed to cycloheximide (CHX, 20 ng/mL). After 0, 3, 6, and 12 h incubation, cells were lysed for protein extraction, and the levels of residual SIRT1 protein were quantified by immunoblotting.

### 2.16 Data Analysis

Intergroup comparisons were conducted using a Student's *t*‐test (unpaired, two‐tailed) or Mann–Whitney *U* test for two‐group analyses. Multigroup comparisons employed one‐way or two‐way ANOVA with Tukey's or Sidak's *post hoc* tests as appropriate. Statistical results were presented as mean ± SD, with the significance threshold established at *p* < 0.05. The diagnostic value of USP28 in clinical specimens was analyzed using the receiver operating characteristic (ROC) curve analysis.

## 3 Results

### 3.1 USP28 Is Upregulated in H1975/OSI Cells and OSI‐Resistant NSCLC Tissues

**FIGURE 1:**
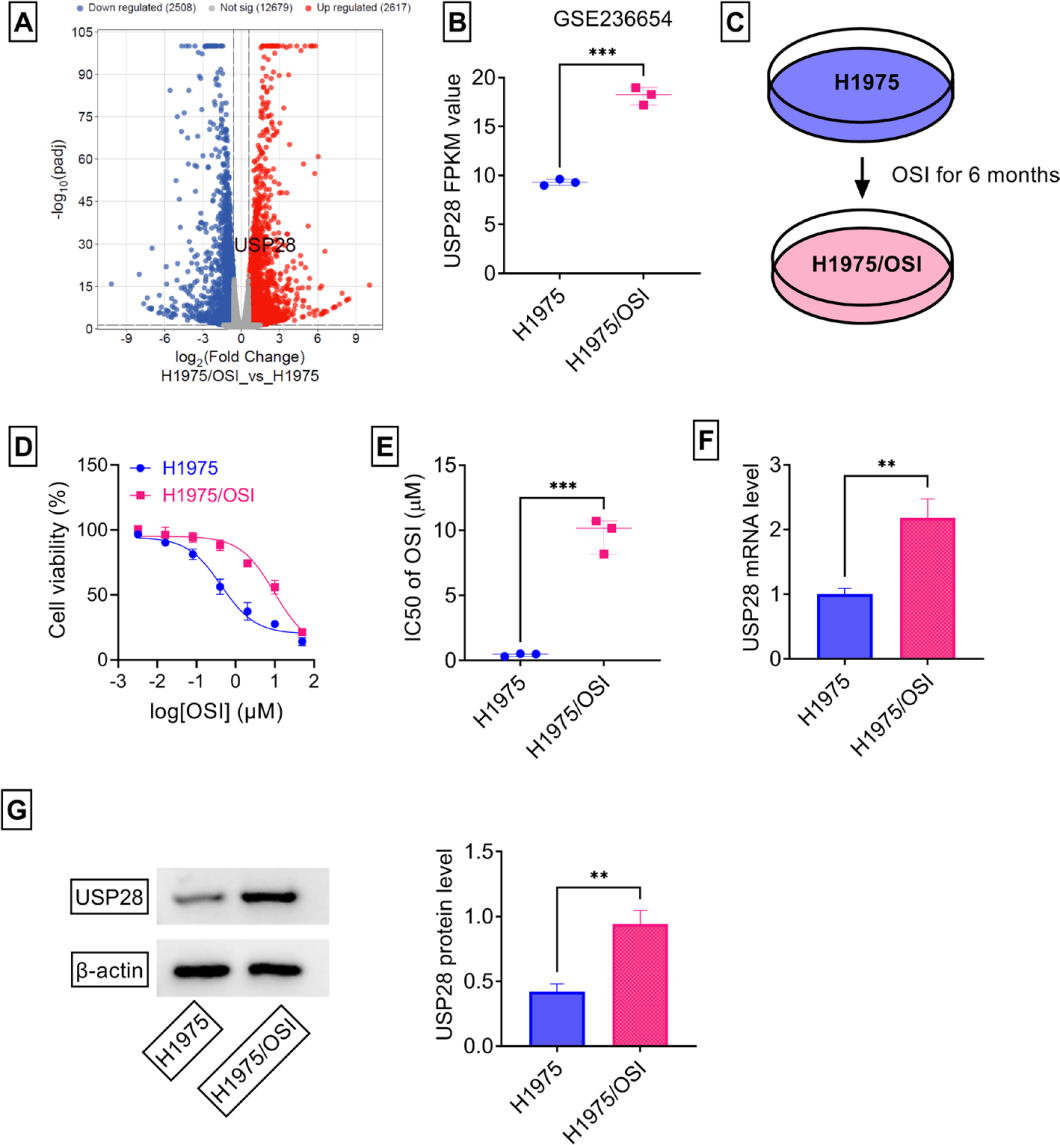
USP28 expression is significantly elevated in H1975/OSI cells. (A) The volcano plot depicting the differentially expressed genes (DEGs) between H1975/OSI cells and H1975 cells using the GSE236654 dataset. (B) The GSE236654 dataset revealing the upregulation of USP28 transcript in H1975/OSI cells. (C) Schematic of the generation of H1975/OSI cells from H1975 cells. (D and E) The dose–response curves for the growth inhibitory effects of OSI in H1975/OSI and H1975 cells assessed by CCK‐8 assay, and their IC50 value of OSI (*n* = 3). (F) Analysis of USP28 mRNA by quantitative PCR in H1975/OSI and H1975 cells (*n* = 3). (G) Analysis of USP28 protein by immunoblot assay in H1975/OSI and H1975 cells (*n* = 3). **p < 0.01, ***p < 0.01.

**FIGURE 2:**
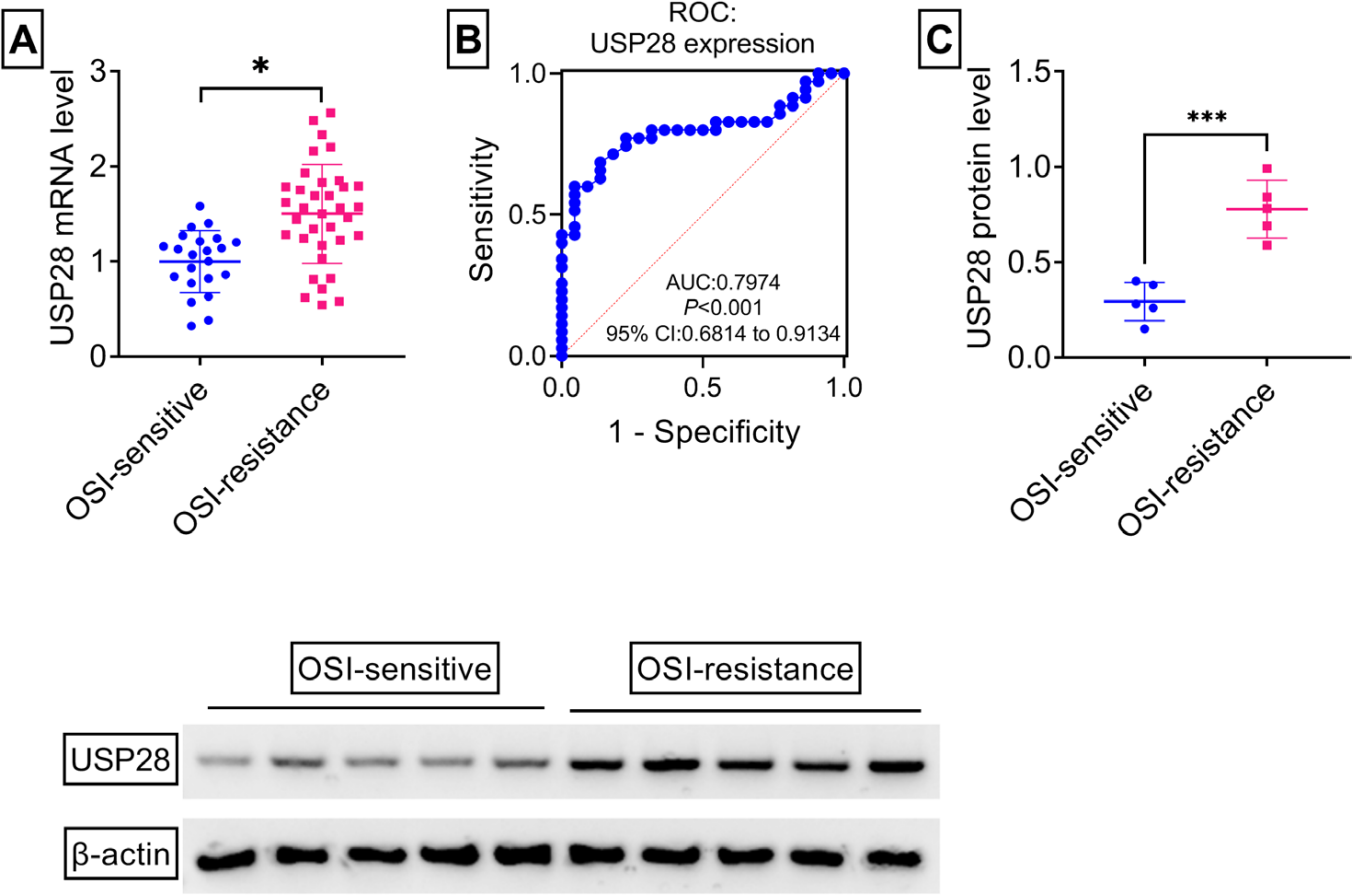
USP28 is upregulated in OSI‐resistant NSCLC tissues. (A–C) Tumor specimens were obtained from two distinct cohorts: 35 recurrent NSCLC patients after treatment based on OSI‐based therapy (resistant group, *n* = 35) and 22 treatment‐naive NSCLC patients who maintained disease control with OSI‐based treatment (sensitive group, *n* = 22). (A) Analysis of USP28 mRNA by quantitative PCR in OSI‐resistant NSCLC samples (*n* = 35) compared with OSI‐sensitive tumor tissues (*n* = 22). (B) The ROC analysis curve of USP28 mRNA expression in these clinical samples. (C) Analysis of USP28 protein by immunoblot assay in OSI‐resistant NSCLC samples (*n* = 5) compared with OSI‐sensitive tumor tissues (*n* = 5). *p < 0.05, ***p < 0.01.

To identify factors associated with OSI resistance in NSCLC, this study analyzed the GSE236654 dataset, which profiles differentially expressed genes (DEGs) between H1975/OSI cells and their sensitive parental counterparts H1975. Applying screening thresholds of *p* < 0.05 and |Fold‐Change| ≥ 1.5, the volcano plot revealed 2617 upregulated genes and 2508 downregulated genes (Figure 1A). Among these DEGs, USP28, a crucial deubiquitinating enzyme, stood out due to its well‐documented pro‐tumorigenic role in NSCLC [18, 25]. As shown in Figure 1B, the GSE236654 dataset showed that H1975/OSI cells displayed a significant increase in USP28 transcript when compared with sensitive H1975 cells. To validate this finding, this study developed the H1975/OSI cell line through the culture of H1975 cells in the presence of a low OSI concentration for 6 months, as illustrated in Figure 1C. CCK‐8 cell viability assays revealed that H1975/OSI cells exhibited enhanced resistance to OSI therapy and an increased IC50 value of OSI compared with the parental H1975 cells (Figure 1D,E), confirming the successful generation of OSI‐resistant H1975 cells. Notably, the mRNA and protein levels of USP28 were significantly increased in H1975/OSI cells compared with the parental counterparts (Figure 1F,G). To elucidate the clinical significance of USP28 in mediating OSI resistance in NSCLC, our study conducted a comparative analysis of USP28 expression patterns between OSI‐resistant and sensitive tumors. To this end, tumor specimens were collected from two distinct cohorts: 35 recurrent NSCLC patients after treatment based on OSI‐based therapy (resistant group) and 22 treatment‐naive NSCLC patients who maintained disease control with OSI‐based treatment (sensitive group). Quantitative PCR results revealed that the mRNA levels of USP28 were markedly augmented in OSI‐resistant NSCLC tumors when compared with the sensitive controls (Figure 2A). Furthermore, the ROC curve analysis demonstrated that USP28 had significant diagnostic potential in differentiating OSI‐resistant NSCLC, with an AUC value of 0.7974 at the optimal cut‐off score of 1.42 (Figure 2B). The sensitivity and specificity of USP28 in OSI‐resistant NSCLC were 60% and 95%, respectively (Figure 2B). Consistently, immunoblot analysis showed that USP28 protein levels were remarkably upregulated in OSI‐resistant NSCLC tumors (Figure 2C). Taken together, these data suggest that USP28 is upregulated during the development of OSI resistance in NSCLC.

### 3.2 USP28 Enhances Cell Proliferation and Glycolysis, Suppresses Apoptosis, and Confers OSI Resistance In Vitro

**FIGURE 3:**
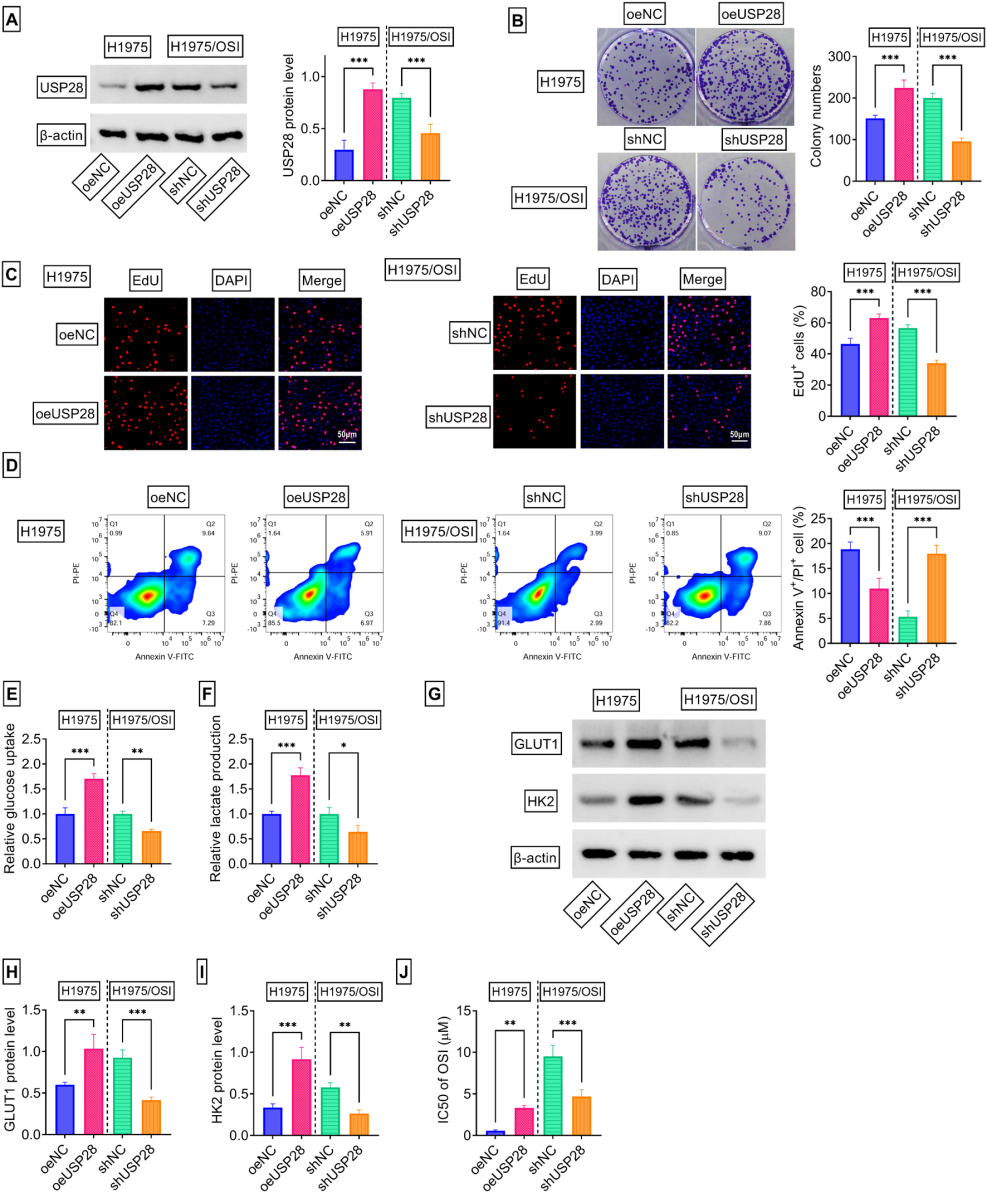
USP28 promotes cell proliferation and glycolysis, inhibits apoptosis, and induces resistance to OSI. (A–J) H1975 cells were subjected to transfection with oeNC or oeUSP28, and H1975/OSI cells were transfected with shNC or shUSP28. (A) Analysis of USP28 protein by immunoblot assay in transfected cells. (B) Representative colony formation assay pictures and the number of generated colonies of cells transfected as indicated. (C) Representative EdU assay images of transfected cells and the ratio of EdU^+^ cells. (D) Analysis of apoptosis in transfected cells by Annexin V and PI staining. (E and F) The levels of glucose uptake and lactate production in cells transfected as indicated using the corresponding assay kit. (G–I) The protein levels of GLUT1 and HK2 in transfected cells evaluated by immunoblot assay. (J) The IC50 value of OSI in transfected cells assessed by CCK‐8 assay. *n* = 3 in (A–J). *p < 0.05, **p < 0.01, ***p < 0.01.

To dissect the functional role of USP28 in mediating NSCLC OSI resistance, our study first examined the functional consequence of increasing USP28 in OSI‐sensitive H1975 cells. A USP28 expression construct (oeUSP28) was employed to elevate USP28 expression, and its efficiency was confirmed to be highly effective in H1975 cells (Figure 3A). USP28 overexpression enhanced the number of generated colonies (Figure 3B) and increased the ratio of EdU^+^ cells (Figure 3C) in H1975 cells. Conversely, increased USP28 levels obviously reduced the percentage of late apoptotic cells (Annexin V^+^/PI^+^) in H1975 cells (Figure 3D). Overexpression of USP28 also augmented the levels of glucose uptake (Figure 3E) and lactate production (Figure 3F) as well as elevated the protein levels of glycolysis‐related factors GLUT1 and HK2 (Figure 3G–I) in H1975 cells. Interestingly, USP28 upregulation led to a dramatic increase in the IC50 value of OSI in H1975 cells (Figure 3J).

Considering the upregulation of USP28 in H1975/OSI cells, this study then elucidated whether reducing USP28 affects cellular phenotypes. As shown in Figure 3A, the protein levels of USP28 decreased upon shUSP28 transfection in H1975/OSI cells. Notably, USP28‐depleted H1975/OSI cells displayed significant growth defects, as evidenced by the reductions in the number of generated colonies (Figure 3B) and the ratio of EdU^+^ cells (Figure 3C) in H1975/OSI cells expressing shUSP28 compared with shNC control cells. Moreover, depletion of USP28 in H1975/OSI cells enhanced cell apoptosis (Figure 3D) and reduced the levels of glucose uptake (Figure 3E), lactate production (Figure 3F), GLUT1, and HK2 (Figure 3G–I) compared with the control group. More intriguingly, USP28 knockdown reduced the IC50 value of OSI in H1975/OSI cells (Figure 3J). Collectively, these results indicate that USP28 overexpression plays a promoting impact on NSCLC progression and OSI resistance development.

### 3.3 USP28 Deubiquitinates and Stabilizes SIRT1

**FIGURE 4:**
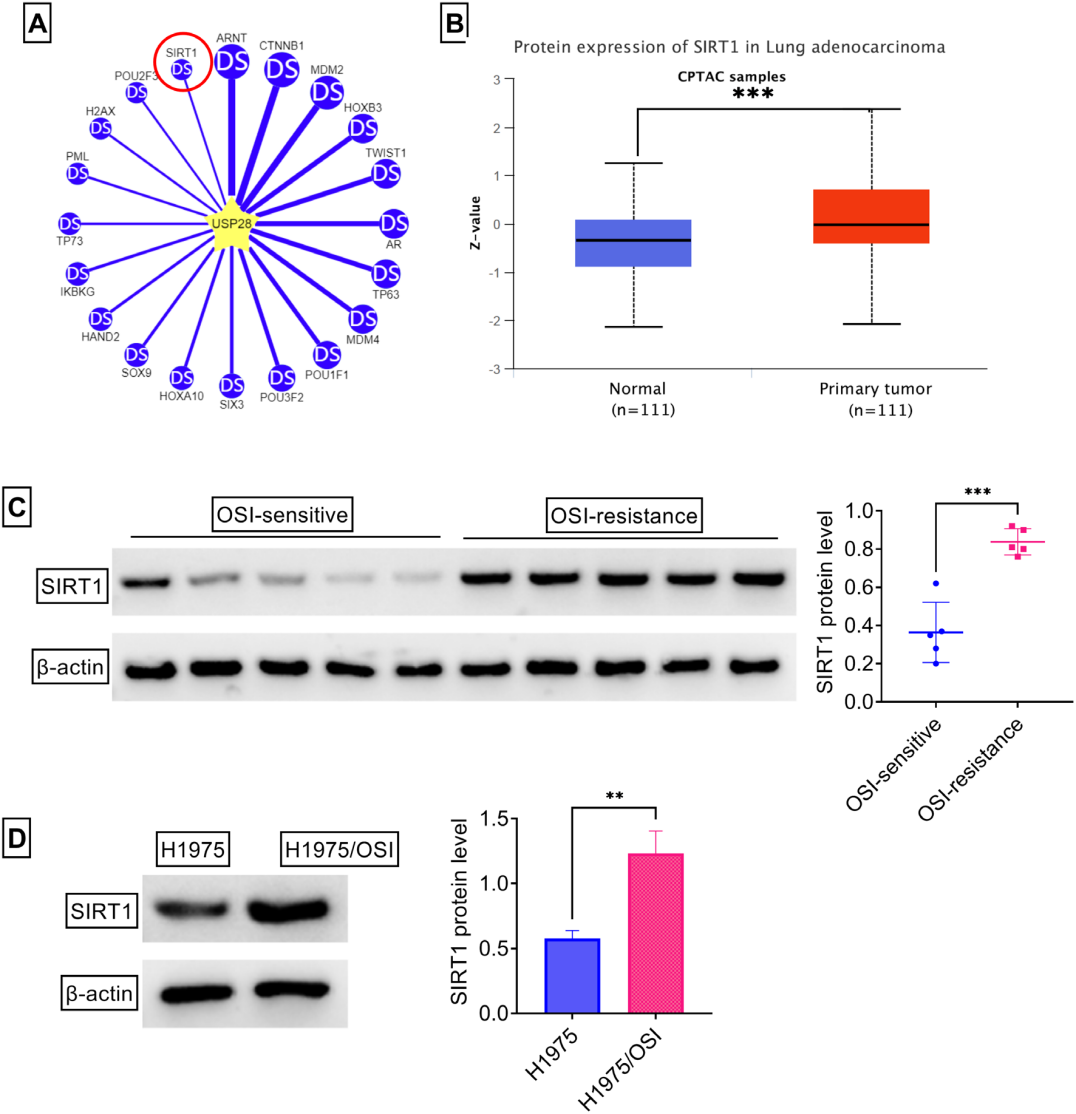
SIRT1 expression is increased in H1975/OSI cells and OSI‐resistant NSCLC tissues. (A) The top 20 potential substrates of USP28 predicted using the Ubibrowser2.0 database. (B) The increased expression of SIRT1 in NSCLC tumors compared with normal counterparts revealed by the UALCAN algorithm. (C) Analysis of SIRT1 protein by immunoblot assay in OSI‐resistant NSCLC samples (*n* = 5) compared with OSI‐sensitive tumor tissues (*n* = 5). (D) Analysis of SIRT1 protein by immunoblot assay in H1975/OSI and H1975 cells (*n* = 3). **p < 0.01, ***p < 0.01.

In order to further search for the downstream effectors of USP28 in the context, this study utilized the online comprehensive Ubibrowser2.0 database to predict its substrates. As shown in Figure 4A, the top 20 potential targets that interacted with USP28 were presented. Among these candidates, SIRT1 was selected since it functions as a potent promoting inducer in NSCLC progression and chemoresistance [26, 27]. The UALCAN algorithm demonstrated the increased expression of SIRT1 in NSCLC tumors compared with normal counterparts (Figure 4B). Indeed, SIRT1 protein levels were significantly overexpressed in OSI‐resistant NSCLC tissues and H1975/OSI cells compared with their sensitive counterparts (Figure 4C,D).

**FIGURE 5:**
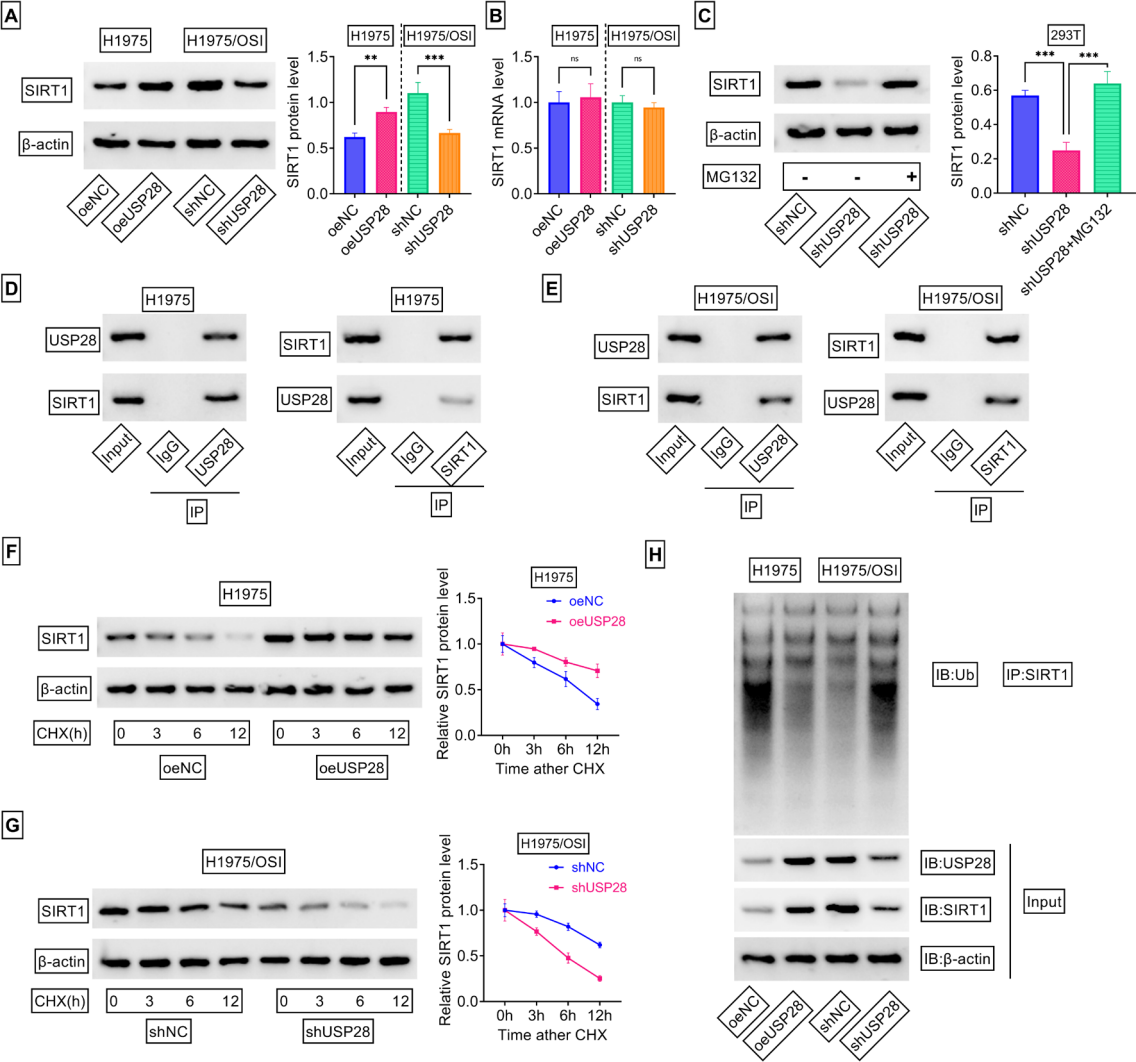
USP28 stabilizes SIRT1 through deubiquitination. (A and B) Analysis of SIRT1 protein by immunoblot assay and SIRT1 mRNA by quantitative PCR in H1975 cells transfected with oeNC or oeUSP28 and H1975/OSI cells transfected with shNC or shUSP28 (*n* = 3). (C) 293 T cells transfected with shNC or shUSP28 were stimulated with or without MG132 (50 μM) and checked for SIRT1 protein levels by immunoblot assay (*n* = 3). (D and E) IP and Co‐IP experiments using an antibody against USP28 or SIRT1, and the subsequent immunoblotting of USP28 and SIRT1. (F and G) Transfected cells were subjected to CHX treatment (50 μg/mL) for the indicated times, followed by detection of SIRT1 protein levels by immunoblot assay (*n* = 3). (H) IP experiments with lysates of transfected cells using an antibody against SIRT1, and the subsequent immunoblotting of ubiquitinated SIRT1 using an antibody against ubiquitin (Ub). **p < 0.01, ***p < 0.01, ns: Non‐significant.

To elucidate the exact role of USP28 in regulating SIRT1, our study first evaluated the modulation in SIRT1 expression. Increased expression of USP28 in H1975 cells led to a striking elevation in SIRT1 protein levels, and USP28 depletion significantly reduced the protein expression of SIRT1 in H1975/OSI cells (Figure 5A), indicating the positive modulation of USP28 in SIRT1 expression. Quantitative PCR results confirmed that USP28 alteration had no obvious impact on SIRT1 mRNA expression (Figure 5B). Moreover, depletion of USP28 in 293 T cells resulted in decreased protein levels of SIRT1, and treatment with MG132, a well‐known inhibitor of ubiquitin‐proteasome, could completely abolish this effect (Figure 5C), suggesting the epigenetic regulation of USP28 in SIRT1 deubiquitination. Our study then examined the interaction between USP28 and SIRT1 in H1975 and H1975/OSI cells. After IP experiments using an antibody recognizing USP28, immunoblot analysis revealed that SIRT1 was detected in USP28‐associated immunoprecipitates (Figure 5D,E). Reciprocally, USP28 was detected in SIRT1 immunoprecipitates (Figure 5D,E). To further understand the influence of USP28 on the stability of SIRT1 protein, USP28‐overexpressing H1975 cells and USP28‐depleted H1975/OSI cells were stimulated with cycloheximide (CHX) for different time points. Under stimulation with CHX to repress protein synthesis, overexpression of USP28 increased the half‐life of SIRT1 protein, and USP28 depletion exhibited an opposite effect (Figure 5F,G), demonstrating that USP28 positively stabilizes SIRT1 protein. Next, this study investigated whether USP28 is indeed able to catalyze the deubiquitination of SIRT1 protein in the two NSCLC cell lines. As illustrated in Figure 5H, the levels of ubiquitinated SIRT1 were markedly decreased after overexpression of USP28. Conversely, the levels of ubiquitinated SIRT1 enhanced upon USP28 depletion in H1975/OSI cells (Figure 5H). Collectively, these findings establish the fact that SIRT1 is a *bona fide* substrate of USP28.

### 3.4 SIRT1 Mediates the Functional Effects of USP28 on Cell Proliferation, Glycolysis, Apoptosis, and OSI Resistance

**FIGURE 6:**
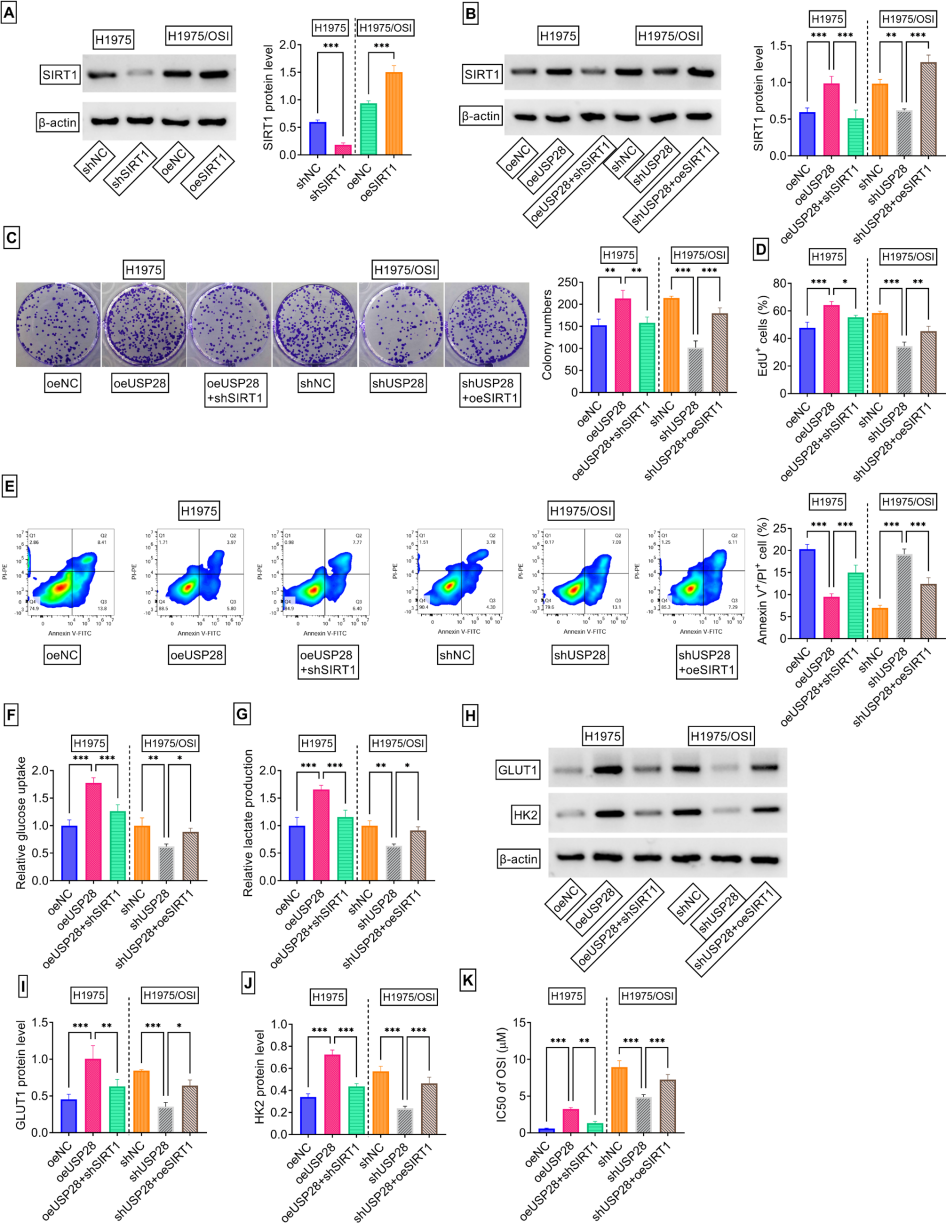
USP28 regulates cell proliferation, glycolysis, apoptosis, and OSI resistance via SIRT1. (A) Analysis of SIRT1 protein by immunoblot assay in transfected cells. (B‐K) H1975 cells were subjected to transfection with oeNC, oeUSP28, or oeUSP28 + shSIRT1, and H1975/OSI cells were transfected with shNC, shUSP28, or shUSP28 + oeSIRT1. (B) Analysis of SIRT1 protein by immunoblot assay in cells transfected as indicated. (C) Representative colony formation assay pictures and the number of generated colonies of transfected cells. (D) The ratio of EdU^+^ cells in transfected cells assessed by EdU assay. (E) Analysis of apoptosis in cells transfected as indicated by Annexin V and PI staining. (F and G) The levels of glucose uptake and lactate production in cells transfected as indicated using the corresponding assay kit. (H‐J) The protein levels of GLUT1 and HK2 in cells transfected as indicated evaluated by immunoblot assay. (K) The IC50 value of OSI in cells transfected as indicated detected by CCK‐8 assay. *n* = 3 in (A–K). *p < 0.05, **p < 0.01, ***p < 0.01.

To delineate the contribution of SIRT1 to the functional effects of USP28 in NSCLC cells, this study conducted rescue experiments. Specifically, SIRT1 expression was reduced in USP28‐overexpressing H1975 cells and conversely, increased SIRT1 levels in USP28‐depleted H1975/OSI cells. As depicted in Figure 6A, immunoblot analysis validated the transfection efficacy of shSIRT1 in reducing SIRT1 expression and oeSIRT1 in upregulating SIRT1 levels. Moreover, shSIRT1 transfection reversed oeUSP28‐driven SIRT1 augmentation in H1975 cells, and oeSIRT1 introduction significantly elevated SIRT1 protein expression in USP28‐depleted H1975/OSI cells (Figure 6B). Functionally, reduction of SIRT1 exhibited a counteracting impact on oeUSP28‐driven promotion in cell colony formation (Figure 6C) and proliferation (Figure 6D), the diminishment in the percentage of late apoptotic cells (Figure 6E), the augmentations in the levels of glucose uptake (Figure 6F), lactate production (Figure 6G), GLUT1, and HK2 (Figure 6H–J) in H1975 cells. SIRT1 reduction also decreased the IC50 value of OSI in H1975 cells overexpressing USP28 (Figure 6K). On the other hand, increased SIRT1 levels significantly abolished USP28 depletion‐mediated colony formation (Figure 6C) and proliferation (Figure 6D) defects, the augmentation in the percentage of late apoptotic cells (Figure 6E), the reductions in the levels of glucose uptake (Figure 6F), lactate production (Figure 6G), GLUT1, and HK2 (Figure 6H–J), as well as the decrease in the IC50 value of OSI (Figure 6K) in H1975/OSI cells. Collectively, these results demonstrate that SIRT1 acts as a downstream effector of USP28 in driving NSCLC progression and OSI resistance development.

### 3.5 Depletion of USP28 Enhances the Therapeutic Efficacy of OSI in H1975/OSI Xenografts In Vivo

**FIGURE 7:**
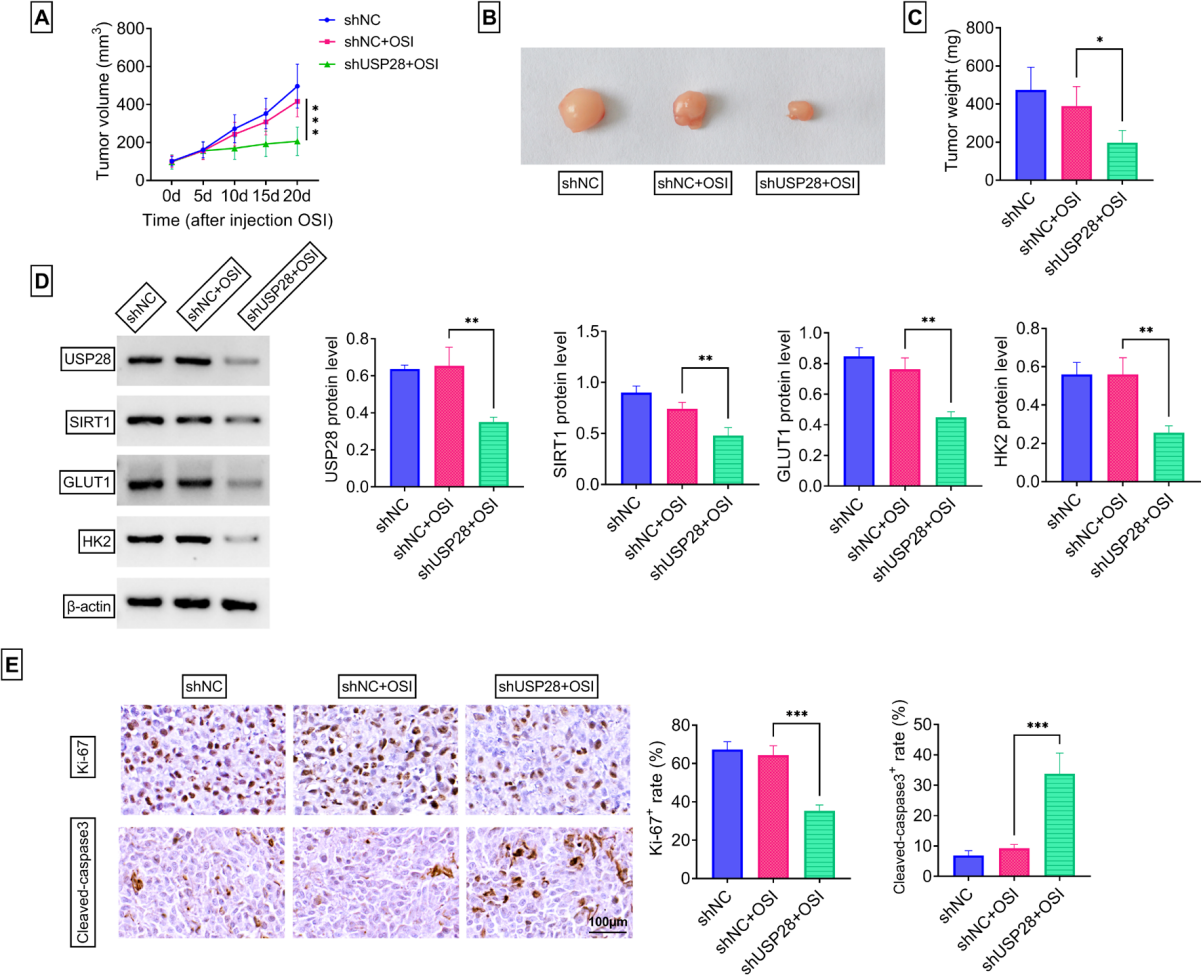
USP28 depletion enhances the efficacy of OSI in suppressing the growth of H1975/OSI xenografts in vivo. (A–E) H1975/OSI cells transduced with shUSP28 or shNC lentivirus were implanted into the right flank through subcutaneous injection and monitored for tumor growth; when tumor volume achieved ~100 mm3, administration of OSI or vehicle began via gavage and performed every 2 days (*n* = 5 for each group). 20 days later, xenograft tumors were harvested. (A) Growth curves of these generated H1975/OSI xenografts. (B) Pictures of these generated H1975/OSI xenografts. (C) Weight measurement of these generated H1975/OSI xenografts. (D) Representative immunoblotting of H1975/OSI xenografts for USP28, SIRT1, GLUT1, and HK2. (E) Representative immunohistochemistry of H1975/OSI xenografts for Ki‐67 and cleaved‐caspase 3. *p < 0.05, **p < 0.01, ***p < 0.01.

These in vitro findings prompted us to test the effect of USP28 downregulation on the therapeutic efficacy of OSI utilizing a subcutaneous H1975/OSI xenograft model. H1975/OSI cells transduced with shUSP28 or shNC lentivirus were implanted into the right flank through subcutaneous injection and monitored for tumor growth; when tumor volume achieved ~100 mm^3^, administration of OSI or vehicle began via gavage and was performed every 2 days. Tumor‐bearing mice receiving shUSP28 lentivirus combined with OSI showed significantly suppressed tumor growth and reduced tumor weight compared with shNC + OSI‐treated mice (Figure 7A–C). Further, immunoblot analysis showed that although the protein levels of USP28, SIRT1, GLUT1, and HK2 were unaffected upon OSI treatment, they significantly decreased in shUSP28 + OSI‐treated xenografts (Figure 7D). Moreover, immunohistochemical analysis revealed that shUSP28 + OSI‐treated xenografts displayed an observable reduction in the proliferation marker Ki‐67 and a striking elevation in the apoptosis‐related protein cleaved‐caspase 3 compared with the shNC + OSI controls (Figure 7E). Collectively, our data suggest that reducing USP28 can enhance the sensitivity of H1975/OSI xenografts to OSI.

## 4 Discussion

Acquired resistance of OSI remains an inevitable challenge, impacting treatment outcomes and patient prognosis [9]. Despite established mechanisms, including tertiary EGFR C797S mutations, MET/HER2 amplification, and phenotypic/histologic transformation, a large proportion of molecular drivers remain undefined [28, 29]. This knowledge gap underscores the urgent need to elucidate novel resistance mechanisms, particularly those involving post‐translational modifications or epigenetic regulators.

Recent studies have highlighted the oncogenic role of USP28 in NSCLC. USP28 inhibition resets the proteome of transformed cells and synergizes with targeted therapies, highlighting its potential for treating early‐stage lung tumors [19]. Moreover, USP28 depletion reduces mevalonate activity and sensitizes NSCLC cancer cells to statins by modulating SREBP2 through deubiquitination [18]. Song et al. also revealed that targeting USP28 induces DNA damage, apoptosis, and immunogenicity via the cGAS‐STING pathway. Combining USP28 inhibition with cisplatin enhances therapeutic efficacy, offering a new strategy to overcome cisplatin resistance in NSCLC [25]. This paper demonstrates that USP28 expression is upregulated in H1975/OSI cells and OSI‐resistant NSCLC tissues. Functionally, USP28 enhances cell proliferation and glycolysis while hindering apoptosis. Importantly, inhibition of USP28 sensitizes H1975/OSI cells to OSI therapy and thus reduces tumor burden in H1975/OSI xenograft models. Thus, our study provides a strong rationale for targeting USP28 as a potential therapeutic way to overcome OSI resistance in patients with advanced NSCLC.

SIRT1 has garnered significant attention in cancer research due to its dual roles in oncogenesis and tumor suppression. SIRT1 is often overexpressed in various cancer types, including colorectal cancer and breast cancer, suggesting its role as a strong oncogenic driver [30, 31]. Conversely, activation of SIRT1 can prevent the development of high‐grade prostatic intraepithelial neoplasia lesions in a Pten−/− mouse model at the pre‐cancer stage, hinting at its potential tumor‐suppressive functions [32]. Recent studies have elucidated the multifaceted roles of SIRT1 in NSCLC, highlighting its context‐dependent functions. For example, Zhu and colleagues have shown that SIRT1 silencing suppresses NSCLC proliferation and induces senescence by modulating p27 stability [26]. In addition, SIRT1 inhibition sensitizes KRAS^Mut^ tumors to cisplatin and erlotinib, revealing a potential therapeutic strategy for KRAS‐driven NSCLC [27]. Conversely, glucose restriction induces the expression of the circadian clock gene Per through the AMPK‐SIRT1 axis, delaying NSCLC progression [33]. Our current study reveals novel insights into SIRT's function in NSCLC, particularly in OSI resistance. Our study shows that SIRT1 levels are increased in OSI‐resistant NSCLC tissues and H1975/OSI cells. Furthermore, our findings identify USP28 as a critical regulator of SIRT1 stability, where USP28 deubiquitinates and stabilizes SIRT1. Importantly, SIRT1 mediates the functional effects of USP28 on cell proliferation, glycolysis, apoptosis, and OSI resistance in NSCLC cells. Our findings uncover a previously unrecognized mechanism by which the USP28/SIRT1 cascade contributes to OSI resistance in NSCLC.

The potential clinical implications of our findings in the realm of NSCLC are significant. First, modulation of the USP28‐SIRT1 axis may offer insights into mitigating the progression of NSCLC, potentially guiding personalized treatment approaches and improving patient prognosis. Preclinical studies have demonstrated that several selective USP28 inhibitors exhibit potent anti‐tumor effects [34]. USP28 inhibitors, such as FT206 and vismodegib, destabilize oncogenic substrates (e.g., c‐Myc, HIF1α) by blocking USP2's deubiquitinase activity, thereby suppressing tumor growth [34]. The USP28‐targeting small molecule CT1113 exhibits anti‐cancer activity by destabilizing oncogenic NOTCH1 and suppressing SREBP1‐mediated lipogenesis in T‐cell acute lymphoblastic leukemia [35]. The USP28 inhibitor AZ1 exerts anti‐tumor effects in NSCLC, and it synergizes with cisplatin to enhance therapeutic efficacy [25]. While clinical translation awaits further safety profiling, these preclinical data strongly support targeting USP28 in OSI‐resistant NSCLC. Furthermore, it may be instructive to consider USP28 inhibition in combination with OSI therapy, opening the possibility for synergistic effects in NSCLC. Several limitations should be acknowledged in our research. First, the novel findings revealed using only one cell line may not be comprehensive, necessitating validation in additional cell lines to ensure robustness. Secondly, the limited number of clinical samples restricts the potential of USP28 as a diagnostic biomarker for OSI‐resistant NSCLC. To address these limitations, future studies should expand the cell line panel and include a larger cohort of clinical samples to validate our observations. Given the context‐dependent functions of SIRT1 in NSCLC, direct inhibition of SIRT1 may yield paradoxical outcomes contingent on the tumor's genetic landscape and microenvironment. For instance, SIRT1 inhibition sensitizes KRAS^Mut^ tumors to cisplatin and erlotinib [27], suggesting therapeutic benefits from its inhibition. However, the AMPK‐SIRT1 axis suppresses NSCLC progression by regulating circadian clock gene Per expression [33]. To address these dichotomies, future strategies should incorporate biomarker stratification (e.g., mutational status and hypoxia signatures), combinatorial approaches with immune checkpoint inhibitors to counter microenvironmental resistance, and development of isoform‐specific inhibitors to preserve tumor‐suppressive SIRT1 functions in normal tissues.

In sum, this study unveils that USP28 can confer OSI resistance in H1975 NSCLC cells by deubiquitinating and stabilizing SIRT1. Our study is the first to elucidate the role of the USP28/SIRT1 axis in the development of OSI resistance in NSCLC. Our findings highlight a novel understanding of the molecular underpinnings of OSI resistance in EGFR‐mutant NSCLC.

## Conflicts of Interest

The authors declare no conflicts of interest.

## Data Availability

The data that support the findings of this study are available from the corresponding author upon reasonable request.
